# Supplementary material for: Uptake of osteoblast-derived extracellular vesicles promotes the differentiation of osteoclasts in the zebrafish scale
Source: Commun Biol. 2020 Apr 23;3:190. doi: 10.1038/s42003-020-0925-1 (PMC7181839; doi:10.1038/s42003-020-0925-1)
Supplement: Supplementary file 1 — Supplementary Information [file 42003_2020_925_MOESM1_ESM.pdf]

Supplementary Figures

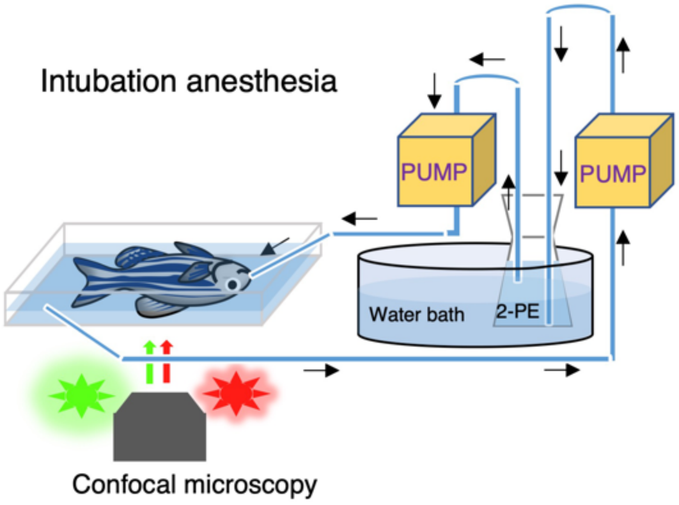

Supplementary Figure 1. Intubation anesthesia system.

A flask containing 2-phenoxyethanol (2-PE) in system water is kept in a water bath to maintain a constant temperature of 28°C, and delivered to a glass-bottom chamber using a peristatic pump. A double-transgenic zebrafish mounted in the chamber is orally perfused with the anesthetic water to image scales.

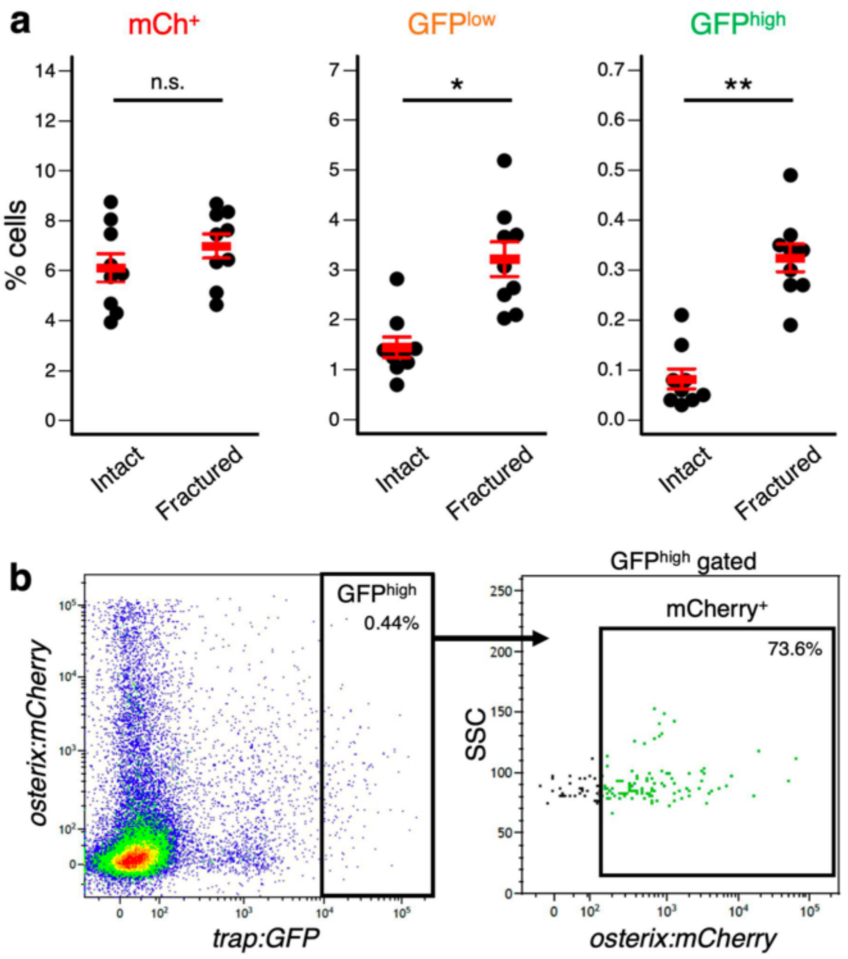

Supplementary Figure 2. OCs increase in the fractured scale.

(**a**) The percentage of *trap:GFP*$^{-}$ *osterix:mCherry*$^{+}$ ("mCh^{+}"), *trap:GFP*^{low} *osterix:mCherry*$^{+}$ ("GFP^{low}"), and *trap:GFP*^{high} ("GFP^{high}") cells in an intact or fractured scale at 1 day post-fracture (dpf). Error bars, s.e.m. (n = 9 for each group); n.s., no significance; *$p < 0.001$; **$p < 0.0001$ by Student's *t*-test. (**b**) Representative flow cytometric analysis of cells in scales at 1 dpf from a *trap:GFP*; *osterix:mCherry* double-transgenic animal. *trap:GFP*^{high} (GFP^{high}) cells in the left panel are displayed in an *osterix:mCherry* vs. side scatter (SSC) dot plot (right panel). Experiments were performed twice with nine biological replicates in each group (**a**, **b**).

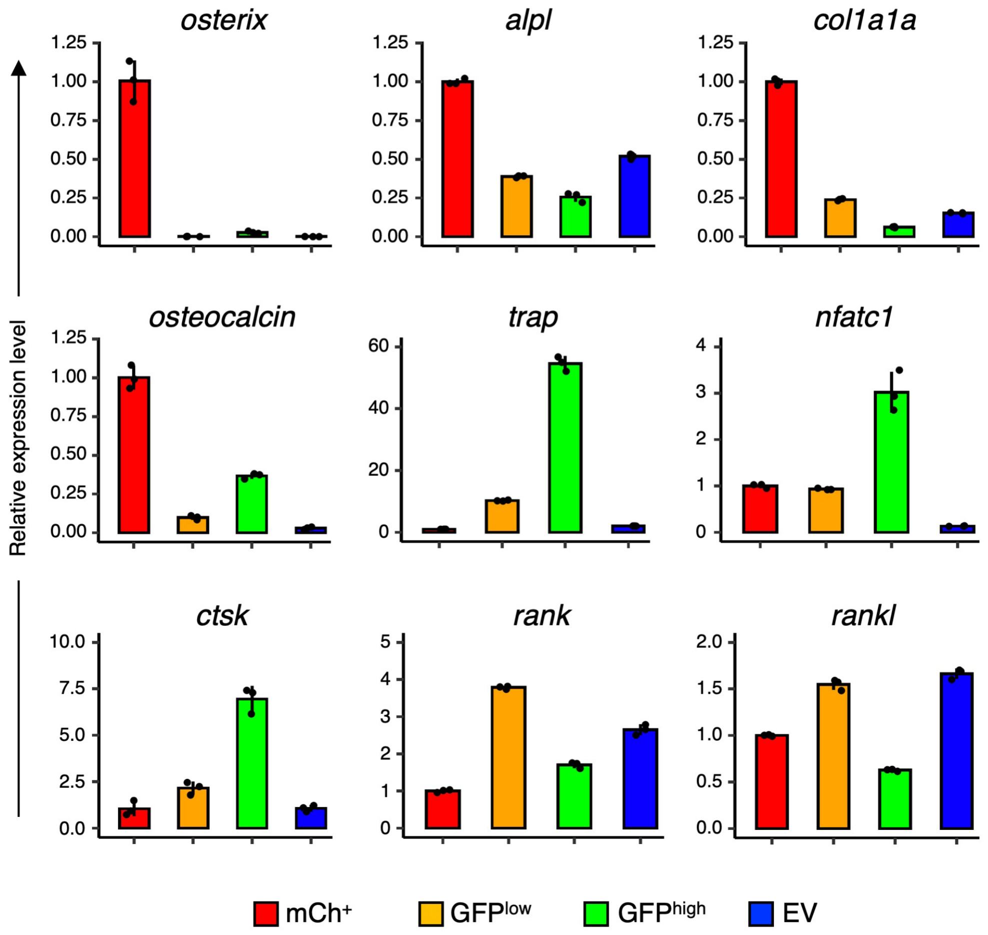

Supplementary Figure 3. Gene expression analysis of OBs, OCs, and OB-derived EVs.

Relative expression levels of *osterix*, *alpl*, *col1a1a*, *osteocalcin*, *trap*, *nfatc1*, *ctsk*, *rank*, and *rankl* in the *trap:GFP*$^{-}$ *osterix:mCherry*$^{+}$ Hoe^{high} ("mCh^{+}"), *trap:GFP*^{low} *osterix:mCherry*$^{+}$ Hoe^{high} ("GFP^{low}"), *trap:GFP*^{high} Hoe^{high} ("GFP^{high}"), and *osterix:mCherry*$^{+}$ Hoe^{low} ("EV") fraction. Data are mean ± s.d. from three independent experiments.

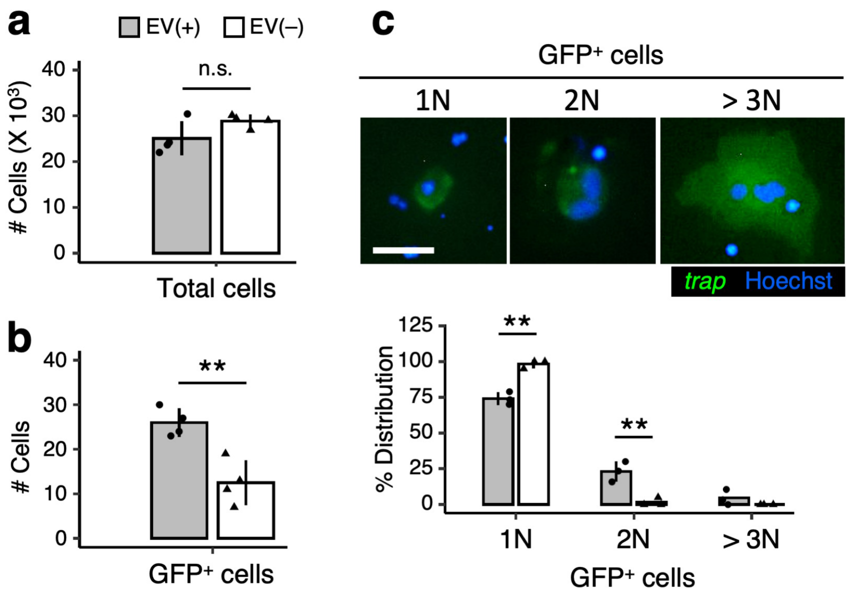

Supplementary Figure 4. Treatment of EVs promotes differentiation and fusion of OCs.
(**a**, **b**) The average number of total cells (**a**) and GFP$^+$ cells (**b**) in the presence or absence of OB-derived EVs. Error bars, s.d. (n = 4 for each group). (**c**) Representative images of *trap:GF*P$^+$ cells co-cultured with EVs (upper panel) and percent distribution of GFP$^+$ cells having a single nucleus (1N) or two (2N) or more than three nuclei (3N) in the presence and absence of EVs (n = 3 for each group). Bar, 20 µm; ***p* < 0.01. Experiments were performed twice with four biological replicates (**a**, **b**) and three biological replicates (**c**) in each group (**a**-**c**).

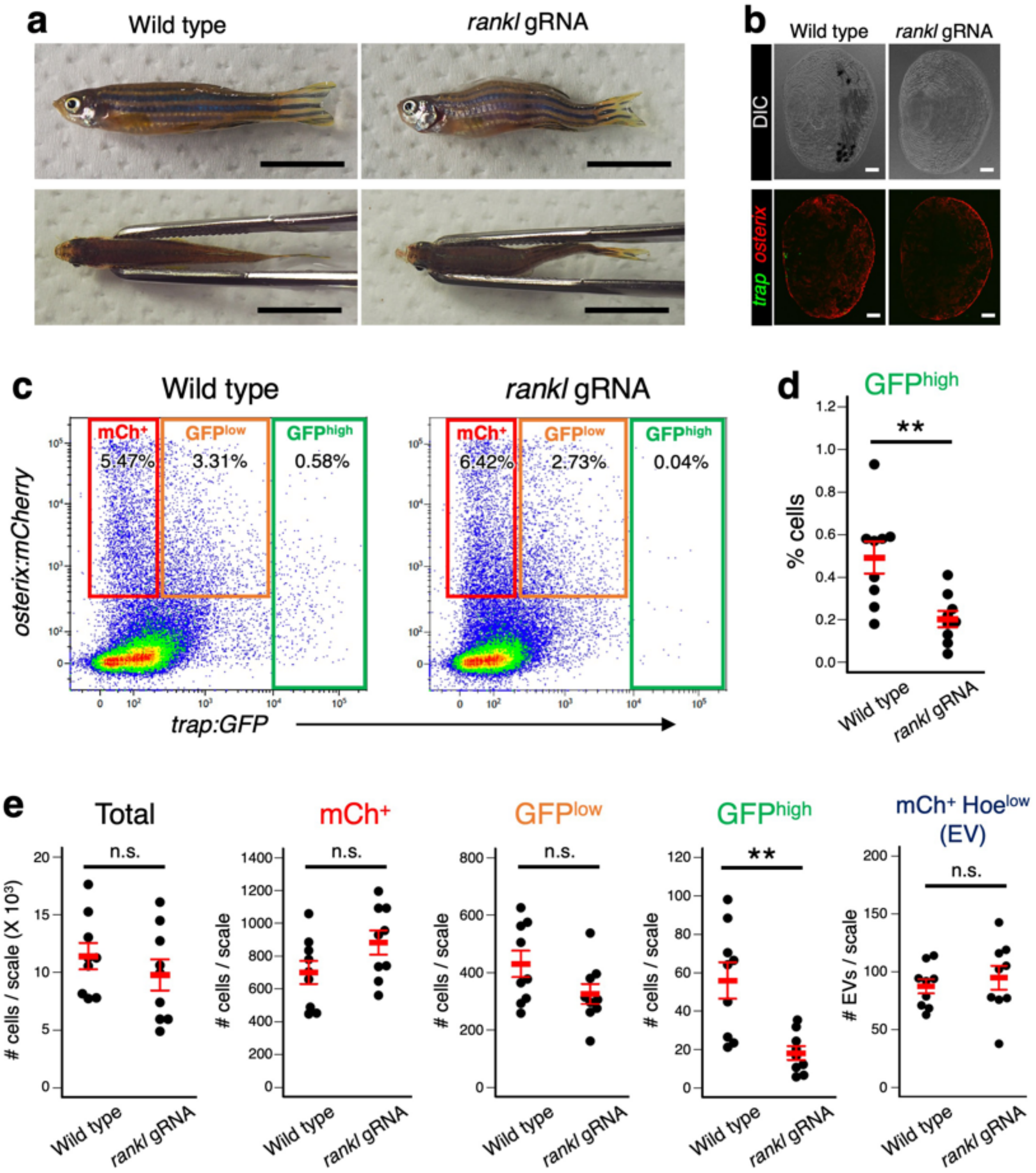

Supplementary Figure 5. *rankl* gRNA-injected zebrafish shows the reduced number of OCs. (**a**, **b**) Representative images of a wild type or *rankl* gRNA-injected zebrafish (**a**) and their scale (**b**) at 4 months of age. *rankl* gRNA-injected zebrafish showed severe body curvature, whereas scales were normally formed. Bars, 1 cm (**a**); 200 µm (**b**). (**c**) Representative flow cytometric analysis of cells in fractured scales at 1 day post-fracture (dpf) from a wild type or *rankl* gRNA-injected zebrafish. Red, orange, and green gate show *trap:GFP*$^{-}$ *osterix:mCherry*$^{+}$ ("mCh^{+}"), *trap:GFP*^{low} *osterix:mCherry*$^{+}$ ("GFP^{low}"), and *trap:GFP*^{high} ("GFP^{high}") cells, respectively. (**d**) Percentage of GFP^{high} cells in fractured scales of wild type or *rankl* gRNA-injected zebrafish at 1 dpf (n = 9 for each group). (**e**) Absolute number of total, mCh^{+}, GFP^{low}, GFP^{high} cells, and mCh^{+} Hoe^{low} EVs in a fractured scale of wild type or *rankl* gRNA-injected zebrafish at 1 dpf. Error bars, s.e.m. (n = 9 for each group); n.s., no significance; *$p < 0.05$; **$p < 0.01$. Experiments were performed twice with nine biological replicates in each group (**c**-**e**).

Supplementary Tables

Supplementary Table 1. Primer and oligo sequences

Gene	Forward primer	Reverse primer	Description
trap (zebrafish enhancer)	CTCGAGGAGATGTAACTTCCAACACTC	GGATCCCCCTACAAAACAACATACAAACAG	Generation of transgenic line
osterix (medaka enhancer)	CTCGAGTGAACATGTCAGTGCCATCAG	GGATCCCGGGACAGTTTGGAAGAAGTC	Generation of transgenic line
ef1a	ACCGGCCATCTGATCTACAA	CAATGGTGATACCACGCTCA	qPCR
osterix	ATTGACCCTCACTGGACTGC	ACCAGGTGTGGCAGAATCTC	qPCR
alpl	GAGAAGCGGCCTGATTACTG	GTCTTAGAGAGGGCGACGTG	qPCR
col1a1a	TTTTGGCAAGAGGACAAGGC	TGTCTTCGCAGATCACTTCG	qPCR
osteocalcin	CTGCTGCCTGATGACTGTGT	TCCAGACGTGTCCATCATGT	qPCR
trap1	ATGATGGCCAAAACTGCTTC	CAGCAATGACGTACCAAGGA	qPCR
nfatc1	TCACTGCCTGCTCTTGATTG	CCTGGTAGAATGCGTGAGGT	qPCR
ctsk	GAGGGAGTACAATGGCCTGA	CCGAAGTGACGTATCCCAGT	qPCR
rank	AATCGCACGGTTATTGTTGTT	ACTGCAGCAAAGTCCCAGTT	qPCR
rankl	TAGTGTGGCGATTCTGTTGC	ATTGGAAGGTGAGCTGATGG	qPCR (primer-1)
rankl	CCATCAGCTCACCTTCCAAT	CGAAAACAGGTCTTGGCGTA	qPCR (primer-2)

Primer sequence for whole-transcript amplification	Description
TATAGAATTCGCGGCCGCTCGCGATAATACGACTCACTATAGGGCGTTTTTTTTTTTTTTTTTTTTTTTT	RT primer
TATAGAATTCGCGGCCGCTCGCGATTTTTTTTTTTTTTTTTTTTTTTTT	Tagging primer
(5' Aminolink)-GTATAGAATTCGCGGCCGCTCGCGAT	Suppression primer

CRISPR/Cas9	Description
TAATACGACTCACTATAGGTGCAGGTCGCGTCTAGTGGTTTTAGAGCTAGAAATAGC	*rankl* target-1
TAATACGACTCACTATAGGTAACCGGTTATCTCCGAGGTTTTAGAGCTAGAAATAGC	*rankl* target-2
TAATACGACTCACTATAGGTATACATAGTAGTATCCAGTTTTAGAGCTAGAAATAGC	*rankl* target-3
TAATACGACTCACTATAGGTCTCATGGTATCGAAAACGTTTTAGAGCTAGAAATAGC	*rankl* target-4
AAAAGCACCGACTCGGTGCCACTTTTTCAAGTTGATAACGGACTAGCCTTATTTTAACTTGCTATTTCTAGCTCTAAAAC	gRNA scaffold primer
