# Supplementary material for: Uptake of osteoblast-derived extracellular vesicles promotes the differentiation of osteoclasts in the zebrafish scale
Source: Commun Biol. 2020 Apr 23;3:190. doi: 10.1038/s42003-020-0925-1 (PMC7181839; doi:10.1038/s42003-020-0925-1)
Supplement: Supplementary file 8 — Description of Additional Supplementary Files [file 42003_2020_925_MOESM8_ESM.pdf]

Description of Additional Supplementary Files

Supplementary Movie Legends

Supplementary Movie 1. Fusion of *trap:GFP*$^{+}$ OCs.

Time-lapse imaging of *trap:GFP*$^{+}$ OCs in the fractured scale from 1 day post-fracture. Arrows indicate a *trap:GFP*$^{+}$ OC that will fuse to become a GFP^{bright} OC. Experiments were performed three times with similar results.

Supplementary Movie 2. Convergence of *trap:GFP*$^{+}$ OCs in the fracture site.

Time-lapse imaging of a fractured scale in a *trap:GFP*; *osterix:mCherry* double-transgenic animal from 1.5 days post-fracture. Arrows indicate a *trap:GFP*$^{+}$ OC that will fuse to become a GFP^{bright} OC around the fracture site. Dotted line indicates the fracture site. Experiments were performed three times with similar results.

Supplementary Movie 3. Motility of type-1 and type-2 OCs.

Time-lapse imaging of *trap:GFP*^{high} cells isolated from the fractured scale. A cell in left with a round morphology and three cells in right having protrusions are classified as type-1 and type-2, respectively. Type-2 OCs actively migrate, whereas a type-1 OC does not. Experiments were performed twice with similar results.

Supplementary Movie 4. Motility of type-2 and type-3 OCs.

Time-lapse imaging of *trap:GFP*^{high} cells isolated from the fractured scale. An upper cell having more than three nuclei and a lower cell having some protrusions are classified as type-3 and type-2 OCs, respectively. A type-3 OC settles, whereas a type-2 OC actively migrates. Experiments were performed twice with similar results.

Supplementary Movie 5. Interaction of a *trap:GFP*$^{+}$ OC with *osterix:mCherry*^{bright} OBs in the fracture scale.

Time-lapse imaging of a fractured scale in a *trap:GFP*; *osterix:mCherry* double-transgenic animal from 1 day post-fracture. Arrow indicates a *trap:GFP*$^{+}$ OC. The *trap:GFP*$^{+}$ OC was actively interacting with *osterix:mCherry*^{bright} OBs, resulting in the uptake of an mCherry^{+} EV. Experiments were performed three times with similar results.

Supplementary Movie 6. Uptake of an OB-derived EV in a *trap:GFP*$^{+}$ OC in the fracture scale.

Time-lapse imaging of a fractured scale in a *trap:GFP*; *osterix:mCherry* double-transgenic animal from 1 day post-fracture. Arrow and arrowheads indicate a *trap:GFP*$^{+}$ OC and mCherry^{+} particles. The *trap:GFP*$^{+}$ OC extended the protrusion, engulfed mCherry^{+} particles, and fused with a GFP^{bright} OC. Experiments were performed six times with similar results.

Supplementary Data Legend

Supplementary Data 1

Source data for Fig. 2, 5, and 7 and Supplementary Fig. 3, 4, and 5.
