# Supplementary material for: Uptake of osteoblast-derived extracellular vesicles promotes the differentiation of osteoclasts in the zebrafish scale
Source: Commun Biol. 2020 Apr 23;3:190. doi: 10.1038/s42003-020-0925-1 (PMC7181839; doi:10.1038/s42003-020-0925-1)
Supplement: Supplementary file 11 — Reporting Summary [file 42003_2020_925_MOESM11_ESM.pdf]

nature research

Corresponding author(s): Isao Kobayashi

Last updated by author(s): Mar 13, 2020

Reporting Summary

Nature Research wishes to improve the reproducibility of the work that we publish. This form provides structure for consistency and transparency in reporting. For further information on Nature Research policies, see Authors & Referees and the Editorial Policy Checklist.

Statistics

For all statistical analyses, confirm that the following items are present in the figure legend, table legend, main text, or Methods section.

n/a	Confirmed	
☐	☒	The exact sample size (*n*) for each experimental group/condition, given as a discrete number and unit of measurement
☐	☒	A statement on whether measurements were taken from distinct samples or whether the same sample was measured repeatedly
☐	☒	The statistical test(s) used AND whether they are one- or two-sided<br>*Only common tests should be described solely by name; describe more complex techniques in the Methods section.*
☒	☐	A description of all covariates tested
☒	☐	A description of any assumptions or corrections, such as tests of normality and adjustment for multiple comparisons
☐	☒	A full description of the statistical parameters including central tendency (e.g. means) or other basic estimates (e.g. regression coefficient) AND variation (e.g. standard deviation) or associated estimates of uncertainty (e.g. confidence intervals)
☐	☒	For null hypothesis testing, the test statistic (e.g. *F*, *t*, *r*) with confidence intervals, effect sizes, degrees of freedom and *P* value noted<br>*Give P values as exact values whenever suitable.*
☒	☐	For Bayesian analysis, information on the choice of priors and Markov chain Monte Carlo settings
☒	☐	For hierarchical and complex designs, identification of the appropriate level for tests and full reporting of outcomes
☒	☐	Estimates of effect sizes (e.g. Cohen's *d*, Pearson's *r*), indicating how they were calculated

Our web collection on statistics for biologists contains articles on many of the points above.

Software and code

Policy information about availability of computer code

Data collection	Fluorescent images were captured using an FV10i confocal microscope and Fluoview FV10i-SW software (ver. 2.1.1) (Olympus). Visible light imaging of adult animals were captured using an Olympus Pen E-PL8 distal camera (Olympus). Electron microscopy images were taken under H-7650 (Hitachi).
Data analysis	We used R (ver. 3.5.0) for statistical analyses.

For manuscripts utilizing custom algorithms or software that are central to the research but not yet described in published literature, software must be made available to editors/reviewers. We strongly encourage code deposition in a community repository (e.g. GitHub). See the Nature Research guidelines for submitting code & software for further information.

Data

Policy information about availability of data

All manuscripts must include a data availability statement. This statement should provide the following information, where applicable:

- Accession codes, unique identifiers, or web links for publicly available datasets
- A list of figures that have associated raw data
- A description of any restrictions on data availability

RNA-seq data have been deposited in Gene Expression Omnibus (GEO) (National Center for Biotechnology Information) and are accessible through the GEO database (series accession number, GSE134330). Source data for main and supplementary figures have been provided in the manuscript. Statistics source data have been provided as Supplementary Data 1. All other data supporting the findings of this study are available from the corresponding author upon reasonable request.

Field-specific reporting

Please select the one below that is the best fit for your research. If you are not sure, read the appropriate sections before making your selection.

☒ Life sciences ☐ Behavioural & social sciences ☐ Ecological, evolutionary & environmental sciences

For a reference copy of the document with all sections, see nature.com/documents/nr-reporting-summary-flat.pdf

Life sciences study design

All studies must disclose on these points even when the disclosure is negative.

Sample size	Sample sizes were chosen after estimating effect size and at least n = 3.
Data exclusions	No data were excluded.
Replication	To ensure reproducibility, data were obtained at least two independent experiments with multiple samples.
Randomization	All experiments comparing treatment groups were made using randomly assigned siblings.
Blinding	n/a

Reporting for specific materials, systems and methods

We require information from authors about some types of materials, experimental systems and methods used in many studies. Here, indicate whether each material, system or method listed is relevant to your study. If you are not sure if a list item applies to your research, read the appropriate section before selecting a response.

Materials & experimental systems

n/a	Involved in the study	
☒	☐	Antibodies
☒	☐	Eukaryotic cell lines
☒	☐	Palaeontology
☐	☒	Animals and other organisms
☒	☐	Human research participants
☒	☐	Clinical data

Methods

n/a	Involved in the study	
☒	☐	ChIP-seq
☐	☒	Flow cytometry
☒	☐	MRI-based neuroimaging

Animals and other organisms

Policy information about studies involving animals; ARRIVE guidelines recommended for reporting animal research

Laboratory animals	Zebrafish (Danio rerio) were used; wild-type AB strain, Tg(trap:GFP-CAAX)ou2031Tg, Tg(osterix:Lifeact-mCherry)ou2032Tg . Three to twelve months of age zebrafish were used in this study. Both male and female animals were used equivalently.
Wild animals	n/a
Field-collected samples	n/a
Ethics oversight	All zebrafish were maintained in accordance with guidelines of the Committee on Animal Experimentation of Kanazawa University.

Note that full information on the approval of the study protocol must also be provided in the manuscript.

Flow Cytometry

Plots

Confirm that:

☒ The axis labels state the marker and fluorochrome used (e.g. CD4-FITC).

☒ The axis scales are clearly visible. Include numbers along axes only for bottom left plot of group (a 'group' is an analysis of identical markers).

☒ All plots are contour plots with outliers or pseudocolor plots.

☒ A numerical value for number of cells or percentage (with statistics) is provided.

Methodology

Sample preparation	All procedures of sample preparation were described in the Materials and methods section of manuscript.
Instrument	FACS Aria III (BD Biosciences)
Software	Kaluza (ver. 1.3, Beckman Coulter)
Cell population abundance	We confirmed that almost all sorted cells were GFP or mCherry-positive by visual inspection using a fluorescent microscope. The numbers for cell sorting were chosen after estimating effect cell number.
Gating strategy	Gating strategies were indicated in each figure.

☒ Tick this box to confirm that a figure exemplifying the gating strategy is provided in the Supplementary Information.
